# Engulfment pathways promote programmed cell death by enhancing the unequal segregation of apoptotic potential

**DOI:** 10.1038/ncomms10126

**Published:** 2015-12-10

**Authors:** Sayantan Chakraborty, Eric J. Lambie, Samik Bindu, Tamara Mikeladze-Dvali, Barbara Conradt

**Affiliations:** 1 Department of Biology II, Ludwig-Maximilians-University, Munich, Center for Integrated Protein Science Munich—CIPSM, LMU Biocenter, Planegg-Martinsried 82152, Germany; 2 Department of Surgery Cardiac & Thoracic Surgery The University of Chicago Biological Sciences, 5841 S. Maryland Ave., Chicago, Illinosis 60637, USA

## Abstract

Components of the conserved engulfment pathways promote programmed cell death in *Caenorhabditis elegans* (*C. elegans*) through an unknown mechanism. Here we report that the phagocytic receptor CED-1 mEGF10 is required for the formation of a dorsal–ventral gradient of CED-3 caspase activity within the mother of a cell programmed to die and an increase in the level of CED-3 protein within its dying daughter. Furthermore, CED-1 becomes enriched on plasma membrane regions of neighbouring cells that appose the dorsal side of the mother, which later forms the dying daughter. Therefore, we propose that components of the engulfment pathways promote programmed cell death by enhancing the polar localization of apoptotic factors in mothers of cells programmed to die and the unequal segregation of apoptotic potential into dying and surviving daughters. Our findings reveal a novel function of the engulfment pathways and provide a better understanding of how apoptosis is initiated during *C. elegans* development.

## Abstract (web summary)

Programed cell death occurs in a stereotypic fashion during *C. elegans* development, and it is thought that engulfment promotes programmed cell death. Here the authors present evidence that a signaling function of the conserved engulfment pathways, not the process of engulfment itself, promotes apoptotic cell death.

## Graphical abstract

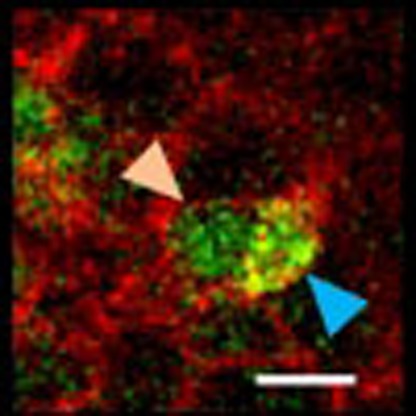

During *Caenorhabditis elegans* (*C. elegans*) development, 131 somatic cells reproducibly undergo programmed cell death. Many of the cells that are programmed to die are the result of an asymmetric cell division and die within 30 min post cytokinesis^1^,^2^. Furthermore, most of the 131 cell deaths are dependent on an evolutionary conserved, central cell death pathway, which is composed of the BH3-only protein EGL-1 (EGL, egg-laying defective), the Bcl-2-like protein CED-9 (CED, cell-death abnormal), the Apaf-1-like adaptor CED-4 and the caspase CED-3 (refs 3, 4, 5). The current model for how the life versus death decision is made during *C. elegans* development is that in cells programmed to live, CED-9 binds to a dimer of CED-4 thereby preventing apoptosome formation and CED-3 maturation. Conversely, in cells programmed to die, EGL-1 is synthesized and binds to CED-9, which results in CED-4 release, apoptosome formation and CED-3 maturation. Active CED-3 subsequently induces processes that are necessary for the regulated dismantling or ‘killing' of the cell and the engulfment and degradation of the resulting cell corpse. For example, CED-3 caspase activates the Xkr8-like protein CED-8, which mediates the exposure of phosphatidylserine (PS) on the surface of the cell corpse^6^,^7^,^8^. Exposed on the cell surface, PS acts as one of the probably several ‘eat me' signals that are recognized by neighbouring cells and that lead to the activation of two conserved, partially redundant engulfment pathways in the neighbouring cells, the CED-1 mEGF10, CED-6 GULP, CED-7 ABC transporter, DYN-1 (DYN, Dynamin related) Dyn1-dependent pathway and the CED-2 CrkII, CED-5 Dock180, CED-10 Rac, CED-12 ELMO-dependent pathway^9^,^10^. The activation of these pathways initiates pseudopod extension, which eventually leads to the complete engulfment and degradation of the cell corpse by one of its neighbours. By recognizing PS on the surface of cell corpses, the mEGF10 (mEGF, multiple epidermal growth factor-like domains)-like receptor CED-1 acts as a phagocytic receptor and plays a critical role in the initiation of this process^11^,^12^,^13^,^14^,^15^. Furthermore, during the engulfment process, CED-1 becomes enriched on those regions of the plasma membrane of the engulfing cell that appose the cell corpse.

The two engulfment pathways also contribute to the actual killing of cells programmed to die. Specifically, it was shown that mutating any component of the two pathways enhances the general cell-death defect (or Ced phenotype) of animals homozygous for weak loss-of-function mutations in *egl-1*, *ced-4* or *ced-3* (refs 16, 17). This ‘killing function' of the engulfment pathways acts in engulfing cells and hence, in a non-cell-autonomous manner. Furthermore, it affects a process or factor that acts downstream of *ced-9* and that is independent of *ced-8* (ref. 17). The mechanism and target of this killing function however, remains to be elucidated.

The two bilaterally symmetric NSM (neurosecretory motorneuron) neuroblasts (NSMnb) are born ∼230 min after the fertilization of the *C. elegans* oocyte (referred to as ‘post-fertilization'). After 180 min (∼410 min post fertilization), they divide asymmetrically and each gives rise to a small cell, the NSM sister cell (NSMsc), and a large cell, the NSM^2^,^18^. The NSM survives and differentiates into a serotonergic motorneuron. The NSMsc, however, undergoes programmed cell death in a manner that is dependent on the central *egl-1*, *ced-9*, *ced-4*, *ced-3*-dependent cell death pathway and forms a corpse within ∼20 min after being generated. Using the apoptotic death of the NSMsc as a paradigm, we present evidence that *ced-1* promotes the death of the NSMsc by contributing to the polar localization of apoptotic factors (including CED-3 caspase activity) within the NSMnb and by enhancing the unequal segregation of apoptotic potential into the NSMsc and NSM. Therefore, our findings reveal a novel function of *ced-1* and other components of the conserved engulfment pathways. Furthermore, within the context of apoptosis, *ced-3* and *ced-1* have been thought to act specifically within the dying cell and within the engulfing cell, respectively. Our findings reveal that in the NSM lineage, *ced-3* and *ced-1* also act earlier, regulating events that occur within the mother of a cell that is programmed to die and within the mother's neighbours. In the context of the NSM lineage, the current model for how apoptotic cell death is initiated therefore needs to be reassessed^3^,^4^,^5^.

## Results

### *ced-1* promotes the death of the NSMsc

**Figure 1:**
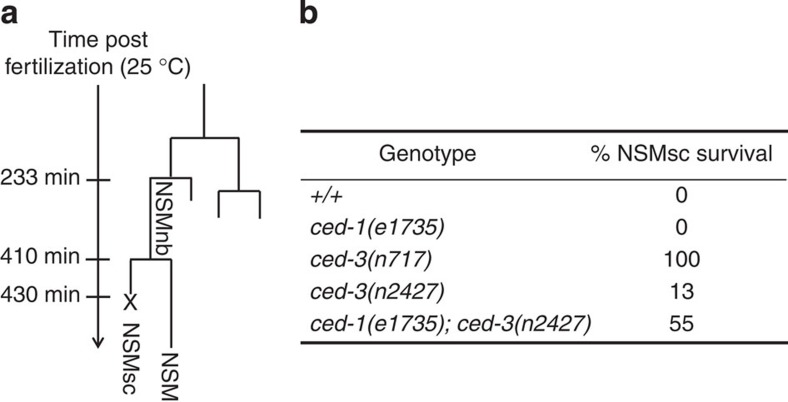
*ced-1* promotes the death of the NSM sister cell. (**a**) NSM lineage depicting the NSM neuroblast (NSMnb), which is born ∼233 min post fertilization (at 25 °C). The NSMnb divides ∼410 min post fertilization to give rise to the NSM, which is programmed to survive, and the NSM sister cell (NSMsc), which is programmed to die at ∼430 min; x indicates the time point of death. (**b**) Percent (%) NSMsc survival scored in L4 larvae in various genetic backgrounds (*n*=60–110). All strains analysed were homozygous for P_*tph-1*_*his-24::gfp* (*bcIs66*).

Using a reporter for serotonergic neurons that is expressed in ‘undead' NSM sister cells^19^,^20^, we investigated whether the *ced-1*, *ced-6*, *ced-7*, *dyn-1*-dependent engulfment pathway promotes the apoptotic death of the NSMsc. We found that a strong *ced-1* loss-of-function mutation (*e1735*) greatly enhances the NSMsc survival phenotype of animals homozygous for *n2427*, a weak loss-of-function mutation in the *ced-3* caspase gene^21^,^22^,^23^ (from 13 to 55%; Fig. 1b). This demonstrates that *ced-1* promotes the death of the NSMsc.

### *ced-1* contributes to increase in CED-3 level in the NSMsc

**Figure 2:**
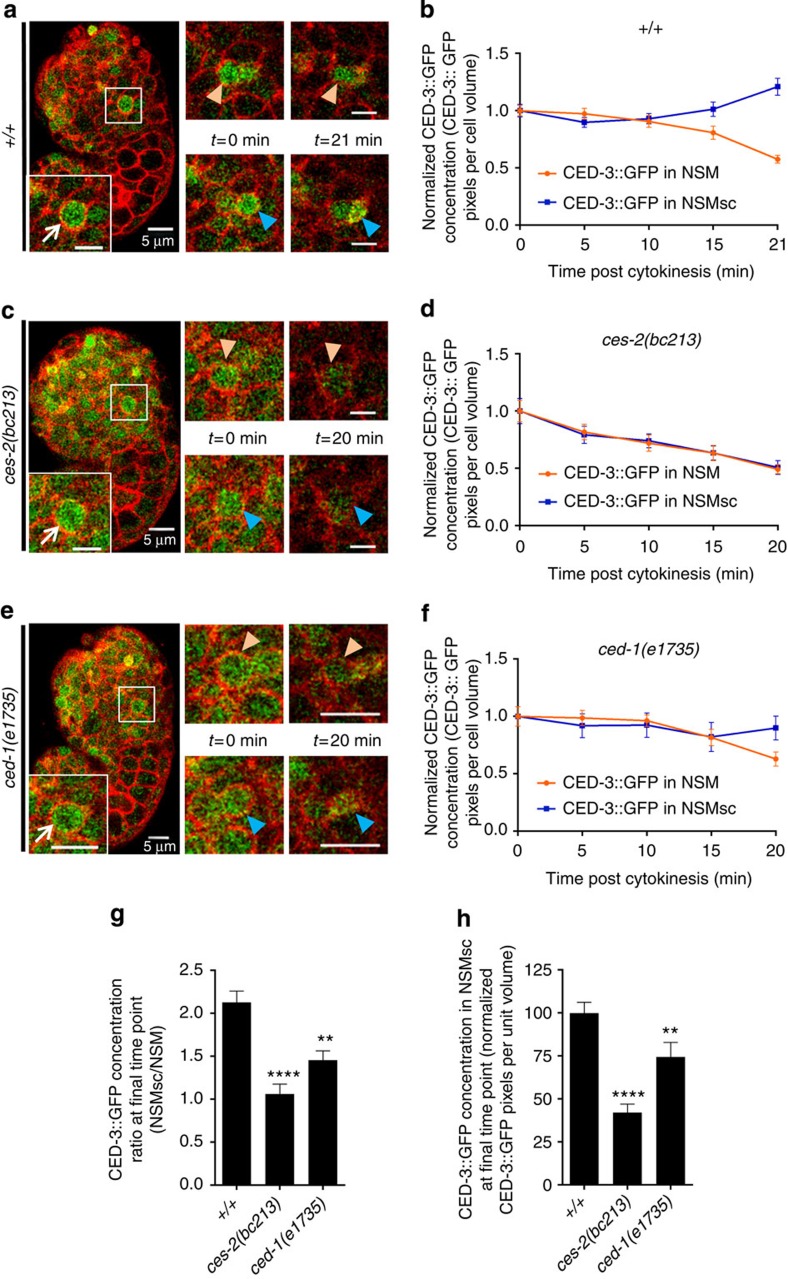
Dynamics of CED-3::GFP in the NSM lineage. (**a**,**c**,**e**) Left images: two-channel overlay projections of single plane confocal images of representative wild type (*+/+*), *ces-2(bc213)* and *ced-1(e1735)* embryos at metaphase expressing P_*ced-3*_*ced-3::gfp* (*bcIs109*) and P_*pie-1*_*mCherry::plcδph* (*ltIs44*). Insets represent enlarged images of NSMnb (scale bar, 5 μm) at metaphase. White arrows point to NSMnb. Right images: single plane confocal images of NSMnb daughter cells at cytokinesis (*t*=0 min) and at the time point at which the NSMsc corpse became visible in *+/+* and *ced-1(e1735)* with the help of mCherry::PLCΔPH (*t*=21 min post cytokinesis for *+/+* and *t*=20 min post cytokinesis for *ced-1(e1735)*; in *ces-2(bc213) t*=20 min was analysed as NSMsc does not form a corpse). Blue arrowheads point to the NSMsc and orange arrowheads point to the NSM. (**b**,**d**,**f**) Normalized means of CED-3::GFP concentration (CED-3::GFP pixels per cell volume) at various time points post cytokinesis (min) in NSMsc (blue) and NSM (orange) in wild type (*+/+*), *ces-2(bc213)* or *ced-1(e1735)*, respectively (*n*=8). (**g**) Ratios of CED-3::GFP concentrations in the NSMsc and in the NSM at final time point. (**h**) Comparison of CED-3::GFP concentrations in the NSMsc at final time point. Statistics were performed using Student's *t*-test by comparing the respective means to wild type (**P≤0.01 and ****P≤0.0001). Error bars denote the s.e.m.

It was previously demonstrated that the killing function of the engulfment pathways affects a factor that acts downstream of *ced-9*, possibly *ced-3* (ref. 17). To investigate when and where *ced-1* could potentially affect *ced-3* in the NSM lineage, we generated a functional, fosmid-based *ced-3* reporter (P_*ced-3*_*ced-3::gfp*) (Supplementary Fig. 1) and analysed its expression in wild-type embryos. We observed ‘CED-3::GFP' (representing both proCED-3::GFP and active CED-3::GFP) in the NSM neuroblast (NSMnb) before its division into the NSMsc and NSM (Fig. 2a; Supplementary Fig. 2). During NSMnb division, CED-3::GFP was equally segregated into the NSMsc and NSM so that the concentrations of CED-3::GFP in the two daughter cells after the completion of cytokinesis were almost identical (*t*=0 min, Fig. 2a and Supplementary Fig. 2). However, starting 10 min post cytokinesis, CED-3::GFP concentration gradually increased in the NSMsc (Fig. 2b). This increase reached a maximum 21 min post cytokinesis, by which time the NSMsc had adopted the morphology typical of a cell corpse (*t*=21 min, Fig. 2a and Supplementary Fig. 2). Conversely, starting 10 min post cytokinesis, CED-3::GFP concentration gradually decreased in the NSM (Fig. 2b). As a result, at 21 min post cytokinesis, the concentration of CED-3::GFP in the NSMsc was more than twofold higher than that in the NSM (Fig. 2b,g).

Next, we asked whether *ced-3* expression within the NSM lineage is affected by a loss-of-function mutation of the HLF (HLF, hepatic leukaemia factor)-like gene *ces-2* (CES, cell-death specification), which affects the asymmetric division of the NSMnb and causes the generation of two cells of similar sizes, both of which survive^18^,^24^,^25^ (Supplementary Fig. 3). We found that in *ces-2(bc213)* animals, the concentration of CED-3::GFP did not increase in the NSMsc post cytokinesis; instead, CED-3::GFP concentration gradually decreased in both the NSM and NSMsc (Fig. 2c,d; Supplementary Fig. 2). As a result, at 20 min post cytokinesis, the concentrations of CED-3::GFP in the NSMsc and NSM were almost identical (Fig. 2d,g). Finally, we analysed *ced-1(e1735)* animals and found that the increase of CED-3::GFP in the NSMsc was delayed compared with wild type (Fig. 2e,f; Supplementary Fig. 2). (As in wild type, in *ced-1(e1735)* animals the division of the NSMnb occurs asymmetrically (Supplementary Fig. 3).) Moreover, the concentration of CED-3::GFP in the NSMsc at the time the NSMsc formed a corpse (20 min post cytokinesis) was significantly reduced (Fig. 2h). As a result, the concentration of CED-3::GFP in the NSMsc at this time point was <1.5-fold higher than that in the NSM (Fig. 2g). (In all genotypes analysed, the changes in NSM and NSMsc volume were comparable (Supplementary Fig. 4).) Based on these observations we conclude that the level of CED-3 caspase increases specifically in the NSMsc and that this increase is at least in part dependent on *ced-1* function.

### CED-1 becomes asymmetrically enriched around the NSMnb

**Figure 3:**
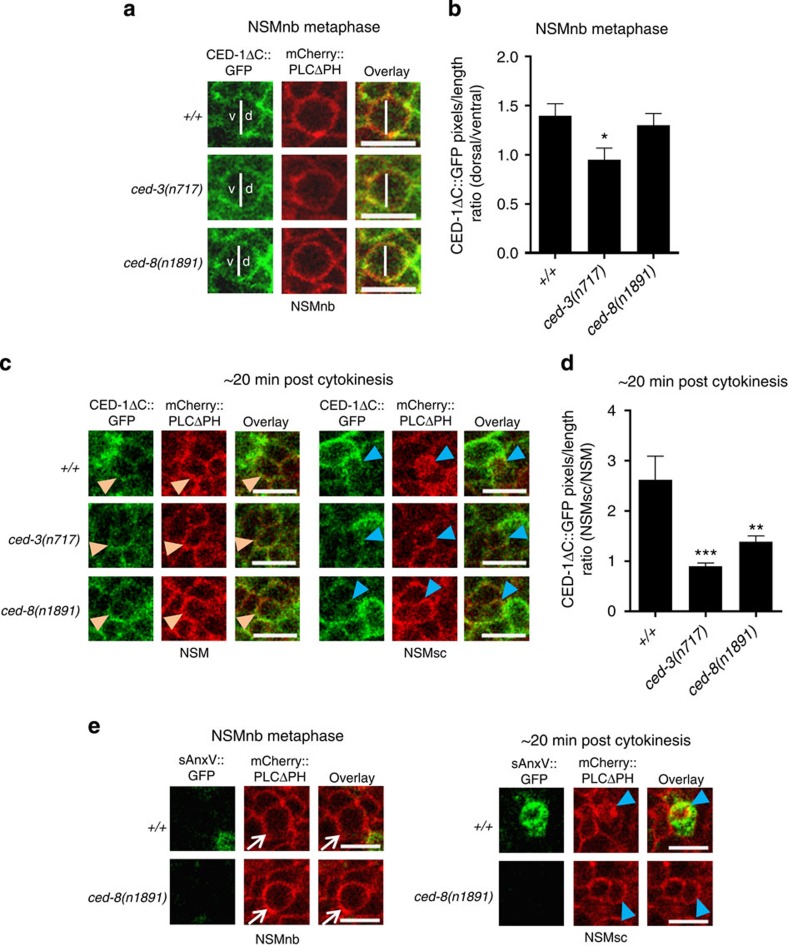
CED-1 becomes enriched in a PS-independent manner on neighbouring cells apposing the dorsal side of the NSMnb. (**a**) Single channel and overlay projections of single plane confocal images of representative NSMnb at metaphase in wild type (*+/+*), *ced-3(n717)* and *ced-8(n1891)* embryos expressing P_*ced-1*_*ced-1δc::gfp* (*enIs1*) and P_*pie-1*_*mCherry::plcδph* (*ltIs44*). White vertical lines indicate the border between the dorsal ‘d' and ventral ‘v' side of the NSMnb (scale bar, 5 μm). (**b**) Ratios of CED-1ΔC::GFP pixels/length apposing the dorsal and the ventral side of the NSMnb at metaphase in various genetic backgrounds (*n*=8–9). (**c**) Single plane and overlay confocal images of representative NSM and NSMsc in wild type (*+/+*), *ced-3(n717)* or *ced-8(n1891)* embryos expressing P_*ced-1*_*ced-1δC::gfp* (*enIs1*) and P_*pie-1*_*mCherry::plcδph* (*ltIs44*) (scale bar, 5 μm). Orange and blue arrowheads denote the NSM and NSMsc, respectively. The red channel has been enhanced for better visualization of the cells. (**d**) Ratios of CED-1ΔC::GFP pixels/length surrounding the NSMsc and the NSM ∼20 min post NSMnb cytokinesis (*n*=4–8). (**e**) Single channel and overlay projections of single plane confocal images of representative NSMnb (at metaphase) and NSMsc (∼20 min post cytokinesis) of representative wild type (*+/+*) and *ced-8(n1891)* embryos expressing P_*hsp*_*sAnxV*::*gfp* (*smIs76*) and P_*pie-1*_*mCherry::plcδph* (*ltIs44*) (scale bar, 5 μm). White arrows depict NSMnb and blue arrowheads depict NSMsc. Statistics were performed using Student's *t*-test by comparing the respective means to wild type (*P≤0.05, **P≤0.01 and ***P≤0.001). Error bars denote the s.e.m.

It was previously demonstrated that the engulfment pathways act in a non-cell-autonomous manner to promote programmed cell death^17^. To determine where and when the CED-1 receptor acts to contribute to the increase in CED-3::GFP in the NSMsc, we used a CED-1::GFP fusion protein (CED-1ΔC::GFP)^13^ to analyse the distribution of CED-1 in neighbours of the NSM lineage. To our surprise, at NSMnb metaphase we found that compared with the ventral side of the NSMnb, CED-1 was enriched 1.3-fold on plasma membrane regions of cells apposing the dorsal side of the NSMnb; this is the side of the NSMnb that later forms the NSMsc (Fig. 3a,b). Enrichment of CED-1 was not observed 5 min before NSMnb metaphase (Supplementary Fig. 5). In addition, it was not observed in animals homozygous for a strong *ced-3* loss-of-function mutation (*n717*) (Fig. 3a,b). As expected, CED-1 was also enriched on those regions of the plasma membranes of engulfing cells that appose the NSMsc corpse (2.5-fold enrichment compared with plasma membrane regions of cells surrounding the NSM; Fig. 3c,d). This enrichment is dependent on *ced-3* as well (Fig. 3c,d).

CED-1 enrichment on plasma membrane regions of engulfing cells is induced and mediated at least in part through PS, which is exhibited on the surface of cell corpses^6^,^8^,^12^. To visualize PS on the surface of cells, we used a secreted AnnexinV::GFP fusion protein (sAnxV::GFP), which binds to PS^26^. We found that PS was present on the surface of the NSMsc but not on the dorsal side of the NSMnb (Fig. 3e). This suggests that differential localization of PS on the NSMnb is not what drives enrichment of CED-1 on the dorsal neighbouring cells. To further test this idea, we used a strong loss-of-function mutation of the gene *ced-8* (*n1891*), which encodes a Xkr8-like protein that is required for PS exposure on the surface of cell corpses^6^,^7^,^8^, including the NSMsc (Fig. 3e). We found that *ced-8*(*n1891*) blocked CED-1 enrichment around the NSMsc, but had no effect on CED-1 enrichment around the dorsal side of the NSMnb (Fig. 3a–d). Hence, we conclude that CED-1 becomes asymmetrically enriched around the dorsal side of the NSMnb and that this enrichment is dependent on *ced-3* function. CED-1 enrichment, however, is independent of *ced-8* and, hence, mediated most probably by a signal other than PS. Interestingly, it was previously shown that the killing function of the engulfment pathways is independent of *ced-8* function^17^.

### Engulfment genes contribute to CED-3 activity gradient

**Figure 4:**
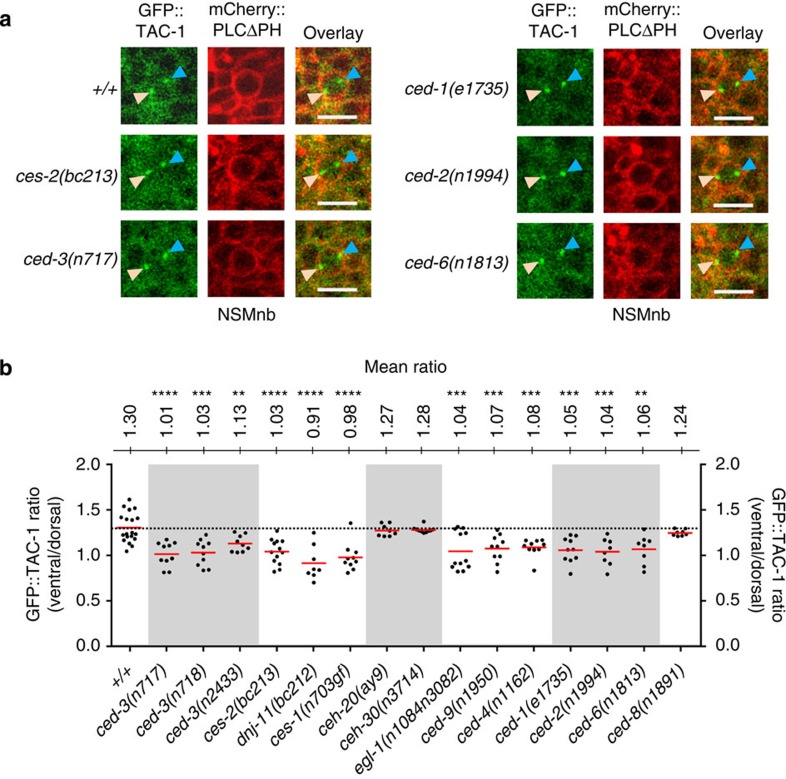
A gradient of CED-3 caspase activity exists in the NSMnb. (**a**) Maximum intensity single channel and overlay projections of confocal images of representative NSMnb at metaphase in wild type (*+/+*), *ces-2(bc213)*, *ced-3(n717)*, *ced-1(e1735)*, *ced-6(n1813)* and *ced-2(n1994)* embryos expressing P_*pie-1*_*gfp::tac-1* (*bcIs104*) and P_*pie-1*_*mCherry::plcδph* (*ltIs44*) (scale bar, 5 μm). Orange arrowheads indicate the PCM in the ventral part of the NSMnb and blue arrowheads indicate the PCM in the dorsal part of the NSMnb. (**b**) Ratios of GFP::TAC-1 pixels associated with the ventral and dorsal PCM in individual NSMnb cells at metaphase in various genetic backgrounds (wild type (*+/+*), *ced-3(n717)*, *ced-3(n718)*, *ced-3(n2433)*, *ces-2(bc213)*, *dnj-11(bc212)*, *ces-1(n703*gf), *ceh-20(ay9)*, *ceh-30(n3714)*, *egl-1(n1084n3082)*, *ced-9(n1950)*, *ced-4(n1162)*, *ced-1(e1735)*, *ced-2(n1994)*, *ced-6(n1813)* and *ced-8(n1891)*) (*n*=8–19). The mean ratio is given (top). For comparison, the dotted line indicates mean ratio of wild type (ratio of 1.3). Mean ratios were analysed using the Student's *t*-test (**P≤0.01, ***P≤0.001 and ****P≤0.0001). All statistical analyses were done in comparisons to wild type.

Our findings demonstrate that *ced-3* is expressed in the NSMnb and *ced-3* function is required for the enrichment of CED-1 on plasma membrane regions of cells apposing the dorsal side of the NSMnb. To visualize CED-3 caspase activity in the NSMnb and to determine its spatial distribution along the dorsal–ventral axis of the NSMnb, we developed an assay based on TAC-1 (TAC, TACC protein family), a component of the pericentriolar material (PCM)^27^,^28^,^29^. TAC-1 has a caspase cleavage site and is a substrate of CED-3 *in vitro* (Supplementary Fig. 6a). When expressing a GFP::TAC-1 fusion protein in the NSMnb, we observed that at metaphase, there is asymmetry in the amount of GFP::TAC-1 that is associated with the PCMs of the two centrosomes. Specifically, compared with the PCM of the centrosome located in the dorsal part of the NSMnb, there was on average 1.3-fold more GFP::TAC-1 associated with the PCM of the centrosome located in the ventral part of the NSMnb (Fig. 4a,b). The loss of *ced-3* (including the *ced-3* active site mutation *n2433*) resulted in an increase in the amount of GFP::TAC-1 associated with the PCM of the centrosome located dorsally (Supplementary Fig. 6b) and thereby disrupted the asymmetry between the amount of GFP::TAC-1 associated with the PCM of the centrosome located in the dorsal and ventral part of the NSMnb (ratios of 1.03 and 1.04, respectively; Fig. 4a,b). Furthermore, a non-cleavable mutant GFP::TAC-1 protein, GFP::TAC-1(D251A), exhibited a ratio in a wild-type background similar to the ratio found for cleavable, wild-type GFP::TAC-1 in a *ced-3* mutant background (ratio of 1.11; Supplementary Fig. 6c,d). Based on these observations we propose that truncated GFP::TAC-1 (GFP-TAC-1Δ C-terminus) that is generated by *ced-3*-dependent cleavage, no longer associates with the PCM. Hence, we infer that the asymmetry between the amount of GFP signal associated with the dorsal and ventral centrosomes of the NSMnb at metaphase reflects a gradient of CED-3 caspase activity within the cell. Higher levels of CED-3 caspase activity are present in the dorsal part of the NSMnb, which subsequently gives rise to the NSMsc.

Next we asked whether this dorsal–ventral gradient of CED-3 caspase activity is dependent on *ces-2* function. We found that in *ces-2* mutants, the gradient is disrupted (ratio of 1.03; Fig. 4a,b). Furthermore, we found that the gradient is also abolished by mutations in *dnj-11* (dnj, DNaJ domain) and *ces-1*, which also disrupt the asymmetric division of the NSMnb and cause the inappropriate survival of the NSMsc^18^ (ratios of 0.91 and 0.98, respectively; Fig. 4b). *dnj-11* encodes a MIDA1 (MIDA, mouse Id associated)/Zrf (Zuotin-related factor)-like chaperone with a potential role in transcriptional control, and *ces-1* encodes a Snail-like Zn-finger transcription factor^18^,^24^,^30^. In addition, we tested whether mutations in *ceh-20* (CEH, *C. elegans* homeobox) and *ceh-30*, which encode transcription factors that control specific cell deaths during *C. elegans* development, but do not regulate cell death within the NSM lineage^31^,^32^,^33^. We found that the gradient of CED-3 caspase activity is not affected by mutations of either *ceh-20* or *ceh-30* (ratios of 1.27 and 1.28, respectively; Fig. 4b). Therefore, the effects of mutations in *ces-2*, *dnj-11* and *ces-1* on the gradient of CED-3 caspase activity in the NSMnb are likely to reflect their specific functions within the NSM lineage, rather than a general consequence of aberrant transcriptional regulation.

We also asked whether the gradient of CED-3 caspase activity within the NSMnb is dependent on components of the central cell death pathway that act downstream of *ces-2* HLF, *dnj-11* Zrf and *ces-1* Snail and upstream of *ced-3*, that is, *egl-1* BH3-only, *ced-9* Bcl-2 and *ced-4* Apaf-1. As shown in Fig. 4b, the gradient is disrupted by mutations in any of these three genes (ratios of 1.04, 1.07 and 1.08, respectively). (Like mutations in *ced-3*, mutations in *egl-1*, *ced-9* and *ced-4* do not affect the asymmetric division of the NSMnb (Supplementary Fig. 3).) This also demonstrates that the central cell death pathway is already active (at least to a certain degree) in the NSMnb.

To our surprise, we also found that the loss of *ced-1* disrupted the gradient of CED-3 caspase activity in the NSMnb (ratio of 1.05; Fig. 4a,b). Similarly, the loss of the CrkII-like adaptor gene *ced-2*, a component of the *ced-2*, *ced-5*, *ced-10*, *ced-12*-dependent engulfment pathway^34^,^35^ or the GULP-like adaptor gene *ced-6*, another component of the *ced-1*, *ced-6*, *ced-7*, *dyn-1*-dependent engulfment pathway^11^,^36^ also disrupted the gradient (ratios of 1.06 and 1.04, respectively; Fig. 4a,b). Therefore, components of both engulfment pathways are also required for the establishment of the dorsal–ventral gradient of CED-3 caspase activity in the NSMnb. Finally, the gradient is not affected by *ced-8*(*n1891*) (ratio of 1.24; Fig. 4b). Since *ced-8* is also not required for the asymmetric enrichment of CED-1 around the NSMnb (Fig. 3a), this finding is consistent with the notion that the asymmetric enrichment of CED-1 around the NSMnb is critical for the generation of the dorsal–ventral gradient of CED-3 caspase activity in the NSMnb.

## Discussion

**Figure 5:**
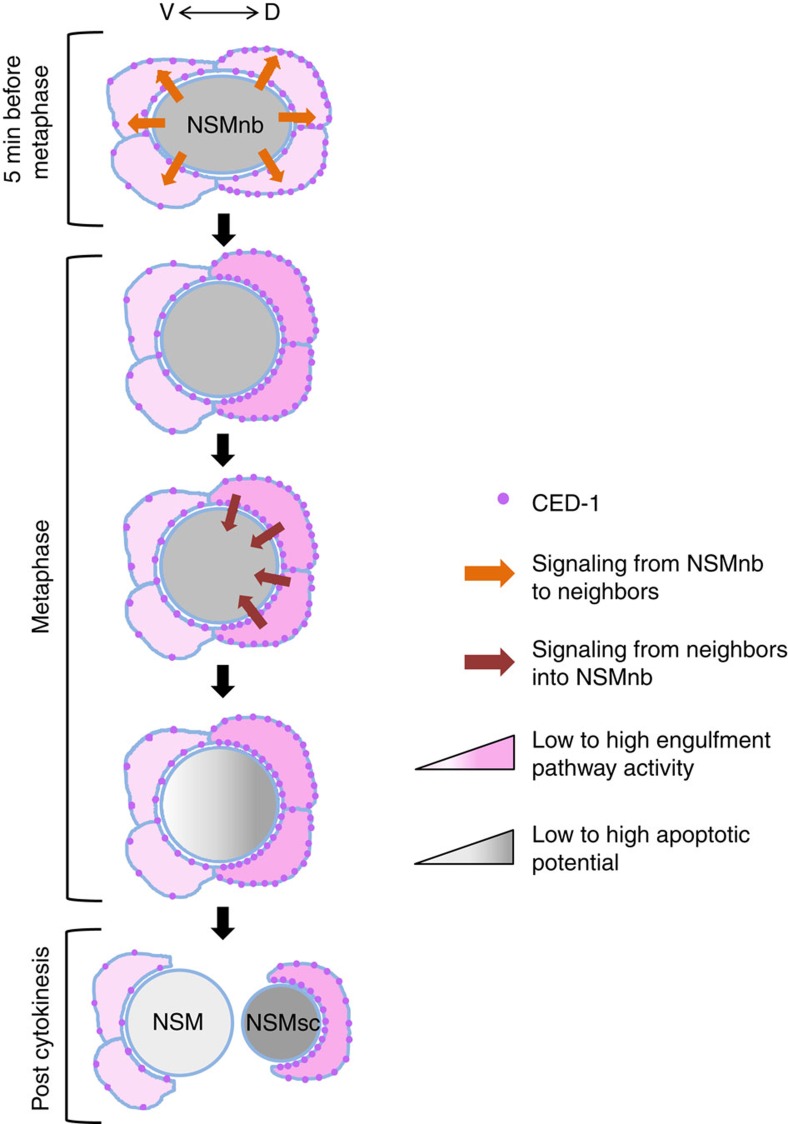
Components of engulfment pathways promote the apoptotic death of the NSMsc. A molecular model for how components of the engulfment pathways promote NSMsc apoptosis (see text for details). CED-3 caspase activity present in the mother of the NSMsc, the NSMnb, produces a signal (orange arrow) that results in the enrichment of CED-1 on plasma membranes of neighbouring cells apposing the dorsal side of the NSMnb. This signal is independent of *ced-8* and PS. We speculate that the dorsal neighbouring cells are primed to receive this signal via a prior symmetry-breaking event, depicted here as somewhat higher expression level of CED-1 in the dorsal neighbours (Supplementary Fig. 7). CED-1 enrichment on the plasma membranes of the dorsal neighbours leads to the activation of components of the two engulfment pathways in these cells. A signal from these neighbours (brown arrow), which is dependent on the functions of components of the two engulfment pathways, subsequently promotes the generation of a gradient of ‘apoptotic potential' (including a gradient of CED-3 caspase activity) along the dorsal–ventral axis of the NSMnb. During NSMnb division, this gradient causes the unequal segregation of apoptotic potential. As a result, the small dorsal daughter cell, the NSMsc, inherits a higher concentration of this potential. This higher concentration is reflected in a higher level of CED-3 caspase activity and an increase in CED-3 concentration in the NSMsc, both of which contribute to the apoptotic death of the NSMsc within ∼20 min post cytokinesis, at which time point the NSMsc corpse is engulfed by a neighbouring cell (Fig. 2a).

Based on our observations, we conclude that it is not the process of engulfment *per se* that promotes the apoptotic death of the NSMsc. Instead, we propose that a previously undescribed signalling function of components of the two engulfment pathways acts in a non-cell-autonomous manner to promote the polarization of the NSMnb and the unequal segregation of apoptotic potential. Specifically, we propose that CED-3 caspase activity, which is already present in the NSMnb (and which is generated in a manner that is dependent on the *egl-1* BH3-only, *ced-9* Bcl-2, *ced-4* Apaf-1 pathway) induces the enrichment of CED-1 mEGF10 in plasma membrane regions of neighbouring cells that appose the dorsal side of the NSMnb. CED-1 enrichment occurs in a *ced-8* Xkr8- and PS-independent manner and results in the subsequent activation of components of the two engulfment pathways in the ‘dorsal' neighbours (Fig. 5). (CED-1 enrichment and activation may occur in dorsal rather than ‘ventral' neighbours since *ced-1* expression in dorsal neighbours appears to be higher than that in ventral neighbours (Supplementary Fig. 7).) This model is consistent with previous findings that suggest that the killing function of the engulfment pathways acts in engulfing cells^17^. However, we cannot completely rule out the possibility that the two engulfment pathways are activated in the dorsal part of the NSMnb, rather than in the dorsal neighbours of the NSMnb. Through a mechanism that remains to be determined, the activation of the engulfment pathways in the dorsal neighbours (or, alternatively, the dorsal part of the NSMnb) then enhances an aspect of NSMnb polarity that is necessary for the establishment of a gradient of apoptotic potential along the dorsal–ventral axis of the NSMnb. During NSMnb division, this gradient results in the unequal segregation of apoptotic potential (Fig. 5). For example, compared with the NSM, more CED-3 caspase activity may segregate into the NSMsc. This notion is supported by the observation that the loss of *ced-3* affects the dissociation of GFP::TAC-1 specifically from the PCM of the dorsal centrosome, which is inherited by the NSMsc (Supplementary Fig. 8). Furthermore, we speculate that gradients of factors other than CED-3 caspase activity are also established, and that these are responsible for the increase in CED-3::GFP observed in the NSMsc and/or the decrease of CED-3::GFP observed in the NSM. For instance, *ced-3* mRNA and/or regulators of *ced-3* mRNA turnover or translation may form gradients and be segregated in an unequal manner into the NSMsc and NSM.

While the loss of this proposed signalling function of the two engulfment pathways blocks the death of only a few cells, its loss in a genetic background in which *ced-3* function is compromised (such as in *ced-3(n2427)*) blocks more than 50% of all programmed cell deaths that occur during *C. elegans* development (Fig. 1b)^16^,^17^. Hence, it is necessary for the robustness of the essentially invariant pattern of programmed cell death observed during *C. elegans* development^1^,^2^. Our observation that the central cell death pathway and the engulfment pathways are active earlier in the NSM lineage than previously anticipated also necessitates a reassessment of how the apoptotic death of the NSMsc is initiated^3^,^4^,^5^,^18^,^20^.

In addition, our findings raise the question of whether apoptotic potential is also segregated in an unequal manner in other cells that divide asymmetrically, such as stem cells, and whether the novel signalling function of components of the engulfment pathways described here is evolutionarily conserved. Previous studies have demonstrated that macrophage-mediated cell killing is an important aspect of mammalian development. For example, the elimination of macrophages during the development of the rat eye results in the inappropriate survival of vascular endothelial cells^37^,^38^. Moreover, resident macrophages of the brain (that is, microglia) promote killing of developing neurons^39^. The molecular mechanisms underlying these phenomena remain unknown. Therefore, it will be interesting to determine whether functional homologues of components of the two *C. elegans* engulfment pathways are functionally involved in the aforementioned mammalian contexts.

## Methods

### Strains

Mutations used in this study are listed below and have been previously described^40^, except where noted otherwise: LGI: *ced-1(e1735)*^34^; *ces-1(n703*gf)^30^; *ces-2(bc213)*^18^. LGII: *bcSi1* [P_*mex-5*_*gfp::tac-1*] and *bcSi4* [P_*mex-5*_*gfp::tac-1(D251A)*] (this study). LGIII: *ceh-20(ay9)*^41^; *ced-9(n1950)* 42; *ced-4(n1162)*^21^,^43^; *bcIs66* [P_*tph-1*_*his-24::gfp*]^18^, *ced-6(n1813)*^11^. LGIV: *ced-2(n1994)*^11^, *ced-3(n717*, *n718*, *n2427*, *n2433)*^22^; *dnj-11(bc212)*^18^. LGV: *egl-1(n1084n3082)*^44^; *enIs1* [P_*ced-1*_*ced-1δc::gfp*]^13^, *ltIs44* [P_*pie-1*_*mCherry::plcδph*]^45^, *smIs76* [P_*hsp*s_*AnxV*::*gfp*]^26^. LGX: *ced-8(n1891)*^11^; *ceh-30(n3714)*^32^. Additional transgenes used in this study were *bcIs109* [P_*ced-3*_*ced-3::gfp*] (this study), *bcIs104* [P_*pie-1*_*gfp::tac-1*]^27^. The strain N2 (Bristol) was used as wild type. Strains were grown on (Nematode growth medium) plates seeded with *Escherichia coli* (*E. coli*) strain OP50 and maintained at 15 °C essentially as described^46^.

### P_*ced-3*_ *ced-3::gfp* fosmid construction

To generate a *ced-3::gfp* reporter a recombineering technique was used^47^ (Supplementary Fig. 1). The fosmid WRM0610cE07 was obtained from Source BioScience UK Limited. It was transformed into *E. coli* strain SW105 followed by a heat shock to induce the expression of the λ Red recombinase. The electro-competent cells containing the fosmid of interest and the recombinase were then allowed to take up a cassette containing the *gfp* sequence as well as the selectable marker *galK*, which is flanked by *frt* sites, the targets of Flp recombinase. The whole cassette was PCR amplified from pBALU1 using the primer sets o1860 (5′-CGCTCATTCAGCAAAGCTTCTGGACCAACTCAATACATATTCCATATGAGTAAAGGAGAAGAACTTTTCAC-3′) and o1861 (5′-AACACGGCTTATGGTTGGTGCATCGACAAAGTTCATATCCTCTTCTTGTATAGTTCATCCATGCCATG-3′) as described^48^. This primer set was designed in a way that the *gfp* tag was inserted at the C-terminal end of *ced-3* in exon 8. Recombinants stably maintaining the *galK* cassette were selected in a minimal medium with galactose. The second recombinase Flipase (Flp) was then induced by the addition of arabinose, thus resulting in the removal of the *galK* marker. Following this, the fosmid (pBC1378) was isolated and electroporated into the *E. coli* strain EPI 300 for amplification and maintenance.

### Germline transformation

Transgenic animals were obtained by microinjecting as described^48^. The P_*ced-3*_*ced-3::gfp* fosmid (pBC1378) (0.5 ng μl^−1^) was co-injected into N2 with the plasmid pRF4 (150 ng μl^−1^), which contains the *rol-6*(*su1006dm*) allele, which causes a dominant Rol phenotype^49^. Three lines carrying extrachromosomal arrays were obtained, one of which was used to stably integrate the array into the genome by ultraviolet radiation. Three integrated lines were obtained. The integrated allele used for our studies was named *bcIs109* and backcrossed twice to wild type. *bcIs109* can restore the wild-type phenotype in a *ced-3(n717)* mutant (Supplementary Fig. 1b).

### TAC-1 biochemistry

The *C. elegans tac-1* cDNA was cloned 3′ of the open-reading frame encoding GST by inserting it into the EcoNI site in the bacterial expression plasmid pGEX-4T-3. The resulting plasmid (pBC1385) was transformed into *E. coli* BL21 (DE3) cells for TAC-1::GST expression. Extraction of TAC-1::GST fusion protein was performed as previously described^50^. Bacteria containing pBC1385 (50 ml culture) were grown to *A*_600_ per ml=0.6 and TAC-1::GST expression induced with 1 mM IPTG for 2 h at 37 °C. Cells were collected, resuspended in 1 ml NETN buffer (20 mM Tris-HCl pH 8.0, 0.5% NP-40, 100 mM NaCl, and a cocktail of protease inhibitor (Roche)) and lysed by sonication. Debris was sedimented by centrifugation and TAC-1::GST fusion protein was purified using 200 μl glutathione agarose beads (GE Healthcare) for 1 h at 4 °C. The beads were washed three times for 10 min with 1 ml of NETN buffer at 4 °C. These beads were subsequently used for the *in vitro* cleavage assay.

CED-3 protein fused to a FLAG octapeptide at its C terminus was obtained from pET-CED-3 plasmid (gift from H. R. Horvitz) expressed in *E. coli* BL21 (DE3) cells. CED-3 lysate was obtained as previously described^51^. The bacteria containing pET-CED-3 was grown in a 50 ml log-phase culture to *A*_600_/ml=0.6 and CED-3 expression was induced with 1 mM IPTG for 2 h at RT. Cells were collected, resuspended in 1 ml of CED-3 buffer (50 mM Tris-HC1 pH 8.0, 0.5 mM EDTA, 0.5 mM sucrose, 5% glycerol and supplementary protease inhibitors) and lysed by sonication. Debris was sedimented by centrifugation and the resulting supernatant was used for the *in vitro* cleavage assay.

For the *in vitro* TAC-1::GST cleavage assay, 20 μl of TAC-1::GST bound glutathione agarose beads (see above) were incubated with 10 μl of CED-3 lysate (see above) and 10 μl of CED-3 buffer (final reaction volume of 40 μl). In parallel, two control reactions were set-up, one without CED-3 lysate and one with CED-3 lysate in which the activity of CED-3 had been inhibited. To inhibit the catalytic activity of CED-3, 10 μl of the CED-3 lysate was pre-incubated for 20 min at 37 °C with 5 mM of an inhibitor (Iodoacetic acid) as described^52^. The cleavage reaction was carried out for 1 h at 37 °C and the reaction was terminated by adding an equal volume of 2 × Laemmli buffer. To analyse the cleavage reaction, a western blot was performed using CeTAC1 (ref. 51) as a primary antibody (1:100) available at Developmental Studies Hybridoma Bank and the horseradish peroxidase-conjugated goat anti-mouse secondary antibody (1:1,000) (Bio-Rad – catalogue #170-6516). The blot was developed using a Chemiluminescence (Amersham) kit and detected using ChemiDoc XRS+ (BioRad).

### GFP::TAC-1 and GFP::TAC-1(D251A) strain construction

*gfp::tac-1* was PCR amplified from *bcIs104*, stitched between the *mex-5* promoter and the *tbb-2* 3'UTR, and cloned into pCFJ350 to generate plasmid pBC1476 [P*mex-5gfp::tac-1*]. To generate a plasmid that expresses CED-3 cleavage-resistant GFP::TAC-1 protein, the codon for aspartate at position 251 of the TAC-1 protein (GAT) was mutated to code for alanine (GCT) and subsequently cloned into pCFJ350 as described above (plasmid pBC1477). Both plasmids were injected separately into *C. elegans* strain EG6699 [*ttTi5605 II; unc-119(ed3) III; oxEx1578*] using the protocol outlined in Wormbuilder (http://www.wormbuilder.org/test-page/protocol/) and integrated into chromosome II^53^. For each plasmid, two independent MosSCI insertions were obtained and named *bcSi1* and *bcSi2* for GFP::TAC-1(+) and *bcSi3* and *bcSi4* for GFP::TAC-1(D215A). *bcSi1* and *bcSi4* were used for analyses.

### Microscopy

*NSMsc survival*. NSM sister-cell survival was scored in L4 larvae or young adults carrying *bcIs66* [P_*tph-1*_*his-24::gfp*] using a Zeiss Axioscop 2 equipped with epifluorescence as previously described^18^.

*Imaging of NSMnb [CED-3::GFP; CED-1ΔC::GFP; sAnxV::GFP and GFP::TAC-1]* Before the experiments, all strains were grown at 25 °C overnight. Adults were dissected to obtain mixed-stage embryos. These embryos were mounted on a 2% agar pad and covered with a glass cover slip that was sealed with petroleum jelly to avoid drying of the embryos. The slides were incubated at 25 °C until the embryos reached the comma stage of development. To induce sAnxV::GFP expression from the heat shock promoter, embryos were incubated at 33 °C for 45 min and subsequently allowed to recover for 2 h as previously described^26^. Imaging was performed using a Leica TCS SP5 II confocal microscope. For all reporters, a Z-stack volume of 7–10 μm with a step size of 0.5 μm was set-up. The recording was initiated before the NSMnb divided and continued until at least 25–30 min post NSMnb cytokinesis for CED-3::GFP, CED-1ΔC::GFP and sAnxV::GFP embryos and at least 10 min post NSMnb cytokinesis for GFP::TAC-1 embryos. Following imaging, a noise reduction step was performed using the Leica Application Suite (LAS) to remove cytoplasmic noise.

*Fluorescence quantifications*. For all reporters, the ventral NSM and dorsal NSMsc were identified by following the division of the NSMnb in each recording.

*CED-3::GFP analysis*. Following confocal acquisition of CED-3::GFP embryos (as described above), for every Z-slice in which a distinct cell boundary (visualized with mCherry::PLCΔPH) for either the NSM or NSMsc could be seen, the number of CED-3::GFP pixels within the cell boundary was determined by drawing a region of interest on the cell boundary. The CED-3::GFP pixels obtained from different Z-slices of a particular cell were summed up to obtain the total CED-3::GFP pixels of that cell. Using the same regions of interest, the volumes of the cells were determined by summing up the areas of the cross sections of each cell. The total number of CED-3::GFP pixels obtained per cell was then divided by the volume of the respective cell and thus the ‘CED-3::GFP pixels/cell volume' or ‘CED-3::GFP concentration' was obtained. This procedure was repeated for all time points analysed in this study. CED-3::GFP concentrations were normalized against the mean CED-3::GFP concentration of the NSM or NSMsc at *t*=0 min. *CED-1ΔC::GFP analysis* (Supplementary Fig. 9). Following confocal acquisition of CED-1ΔC::GFP embryos (as described above), the centre plane of the NSMnb (at metaphase or 5 min before metaphase), the NSM or the NSMsc (both ∼20 min post cytokinesis) was identified and used for analysis. The cell boundaries of NSMnb, NSM and NSMsc were visualized with mCherry::PLCΔPH. The dorsal (‘d') and ventral (‘v') side of the NSMnb was marked based on the position of the NSMnb daughters post cytokinesis. ‘CED-1ΔC::GFP pixels/length' apposing the dorsal or ventral side of the NSMnb was determined by summing up the pixels apposing either side of the cell and dividing it by the length of the respective cell boundary. Post NSMnb cytokinesis (∼20 min post cytokinesis), ‘CED-1ΔC::GFP pixels/length' was obtained by summing up the pixels surrounding the cell boundary of either daughter cell and dividing it by its circumference.

*GFP::TAC-1 analysis (Supplementary Fig. 10)*. Following confocal acquisition of GFP::TAC-1 embryos (as described above), a region of interest with a constant area for all slices of a Z-stack was drawn around the ‘dorsal' or ‘ventral' PCM in the NSMnb at metaphase. The total number of GFP::TAC-1 pixels associated with either PCM through the entire Z-stack was determined by summing up the number of GFP::TAC-1 pixels obtained from the different slices. The mean GFP::TAC-1 pixels of ‘ventral' and ‘dorsal' PCM in wild type were normalized to 1. Each of the ventral and dorsal GFP::TAC-1 pixels obtained for *ces-2(bc213)*, *ced-3(n717)* and *ced-3(n718)* embryo were normalized against this value of 1.

The time of GFP::TAC-1 dissociation (TOD) in the NSMsc was obtained by following the GFP::TAC-1 signal associated with the PCM inherited by the NSMsc from the onset of anaphase (the first image frame at which the centrosomes move away from their metaphase position) until no GFP::TAC-1 signal associated with the PCM was detected anymore.

*NSMsc to NSM cell volume analysis*. On confocal acquisition of CED-3::GFP or GFP::TAC-1 embryos (as described above), a region of interest was drawn for every Z-slice in which a distinct cell boundary (visualized with mCherry::PLCΔPH) for either the NSM or NSMsc could be seen. Using the region of interests, the area of each cell was determined for every Z-slice and then summed up to obtain the total cell volume. The cell volume of the NSMsc was divided by that of the NSM to determine ‘cell volume ratio'. In case of CED-3::GFP embryos, this procedure was repeated for all time points analysed in this study and the cell volumes were normalized against the mean CED-3::GFP concentration of the NSM or NSMsc at *t*=0 min.

## Additional information

**How to cite this article:** Chakraborty, S. *et al*. Engulfment pathways promote programmed cell death by enhancing the unequal segregation of apoptotic potential. *Nat. Commun.* 6:10126 doi: 10.1038/ncomms10126 (2015).

## Supplementary Material

Supplementary Supplementary Figures 1-10
