# Supplementary material for: Engulfment pathways promote programmed cell death by enhancing the unequal segregation of apoptotic potential
Source: Nat Commun. 2015 Dec 10;6:10126. doi: 10.1038/ncomms10126 (PMC4682117; doi:10.1038/ncomms10126)
Supplement: Supplementary — Figures 1-10 [file ncomms10126-s1.pdf]

a

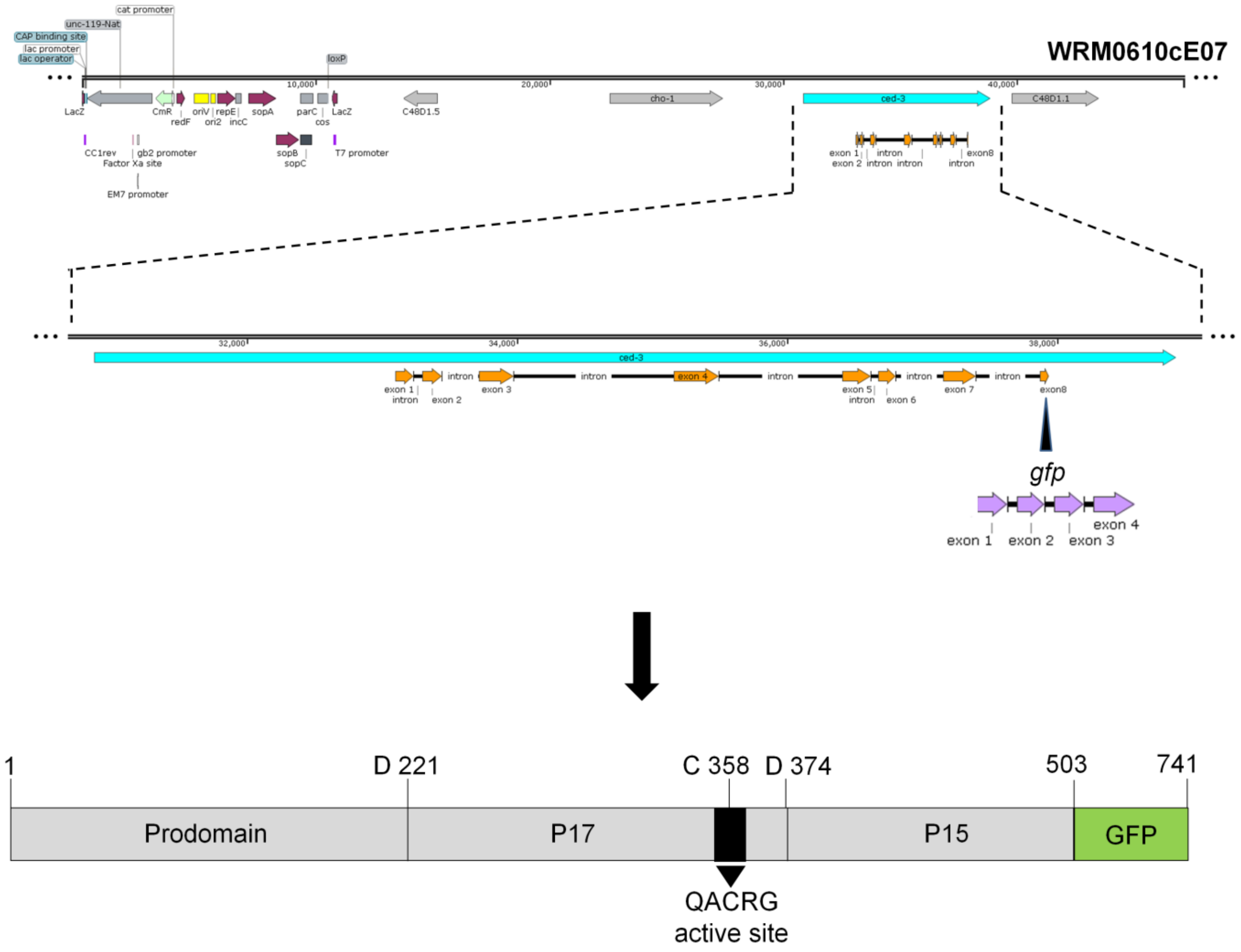

b

Genotype	Number of extra cells in anterior pharynx
+/+	0.0 ± 0.0
ced-3(n717)	11.2 ± 0.37
ced-3(n717); bcIs109	0.4 ± 0.19

Supplementary Figure 1. A fosmid based P$_{ced\text{-}3}$*ced-3::gfp* reporter complements *ced-3(n717)*.

a, Top: Fosmid map of WRM0610cE07 with *ced-3* transcription unit. The complete fosmid map with the gene annotations was obtained from the *C. elegans* TransgeneOme project (https://transgeneome.mpi-cbg.de/transgeneomics/index.html) Middle. Exon-intron structure

of *ced-3* transcription unit. The open reading frame of *gfp* was inserted in frame at the 5' end of exon eight to generate $P_{ced\text{-}3}$*ced-3::gfp* (pBC1378; see Methods – $P_{ced\text{-}3}$*ced-3::gfp* fosmid construction). Upon expression of this construct, both proCED-3 and active CED-3* protein are fused to GFP and can be visualized *in vivo*. Bottom: Schematic of proCED-3::GFP fusion protein. The prodomain, p17 and p15 domain as well as the active site are indicated. Numbers above bar represent amino acid positions. **b,** Number of extra cells in the anterior pharynx for the given genotypes. A stable, integrated transgene of $P_{ced\text{-}3}$*ced-3::gfp* (*bcIs109*) rescues the general cell-death (Ced) defect of *ced-3(n717)* animals. Data are means ± standard error of the mean (n=12-15).

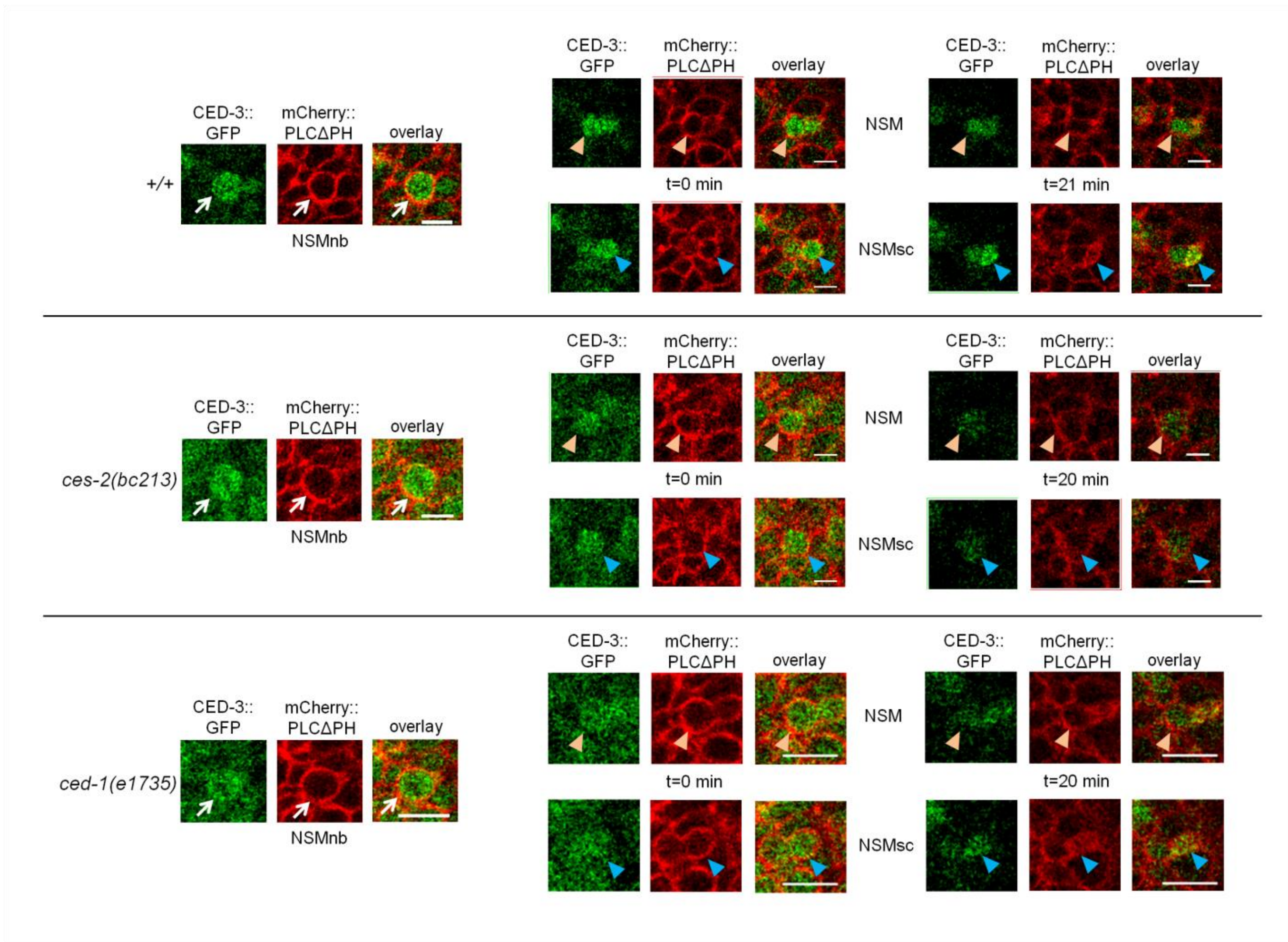

Supplementary Figure 2. CED-3::GFP in the NSM lineage

Single channel and overlay projections of single plane confocal images of the NSMnb at metaphase, NSM and NSMsc at t=0 min and t= 20 or 21 min post cytokinesis, respectively, in wild-type (+/+), *ces-2(bc213)* and *ced-1(e1735)* embryos expressing $\mathrm{P}_{ced\text{-}3}$*ced-3::gfp* (*bcIs109*) and $\mathrm{P}_{pie\text{-}1}$*mCherry::plcδph* (*ltIs44*) (scale bar = 5 µm). White arrows point to NSMnb. Blue arrowheads point to the NSMsc and orange arrowheads point to the NSM.

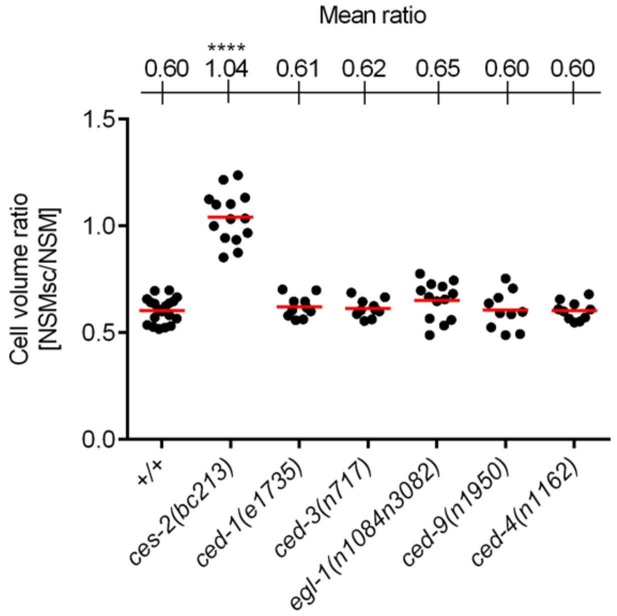

Supplementary Figure 3. Like in the wild-type, in *ced-1* and *ced-3* mutant animals, the NSMnb divides asymmetrically to give rise to a small and large daughter cell.

Ratio of the NSMsc cell volume to the NSM cell volume post cytokinesis of individual NSMnb divisions in different genetic backgrounds (wild-type (+/+), *ces-2(bc213), ced-1(e1735)*, *ced-3(n717)*, e*gl-1(n1084n3082)*, *ced-9(n1950)*, *ced-4(n1162)*) expressing $P_{pie\text{-}1}$*gfp::tac-1* (*bcIs104*) and $P_{pie\text{-}1}$*mCherry::plcδph* (*ltIs44*) (n=10-19). Mean ratios are indicated on top. All statistical analyses (Student's t-test) were done in comparison to wild-type (+/+) (****p≤0.0001).

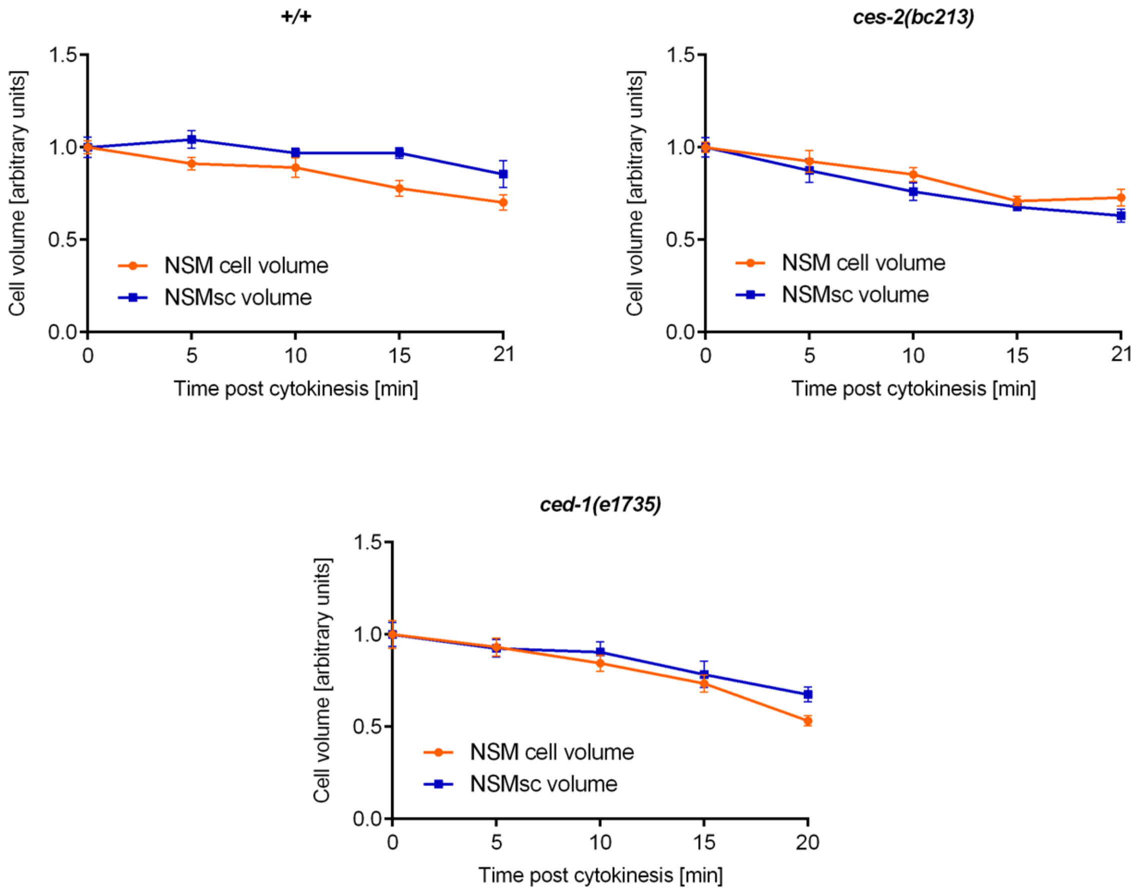

Supplementary Figure 4. Changes in NSM and NSMsc volume post NSMnb cytokinesis

Cell volume analysis of NSM and NSMsc post NSMnb cytokinesis in embryos at different time points expressing $P_{ced\text{-}3}$*ced-3::gfp* (*bcIs109*) and $P_{pie\text{-}1}$*mCherry::plcδph* (*ltIs44*) in the given genetic backgrounds (wild-type (+/+), *ces-2(bc213)* and *ced-1(e1735)*) (n=8). All cell volumes obtained for NSM or NSMsc at the various time points were normalized against the cell volume obtained at t=0 min for the respective cell.

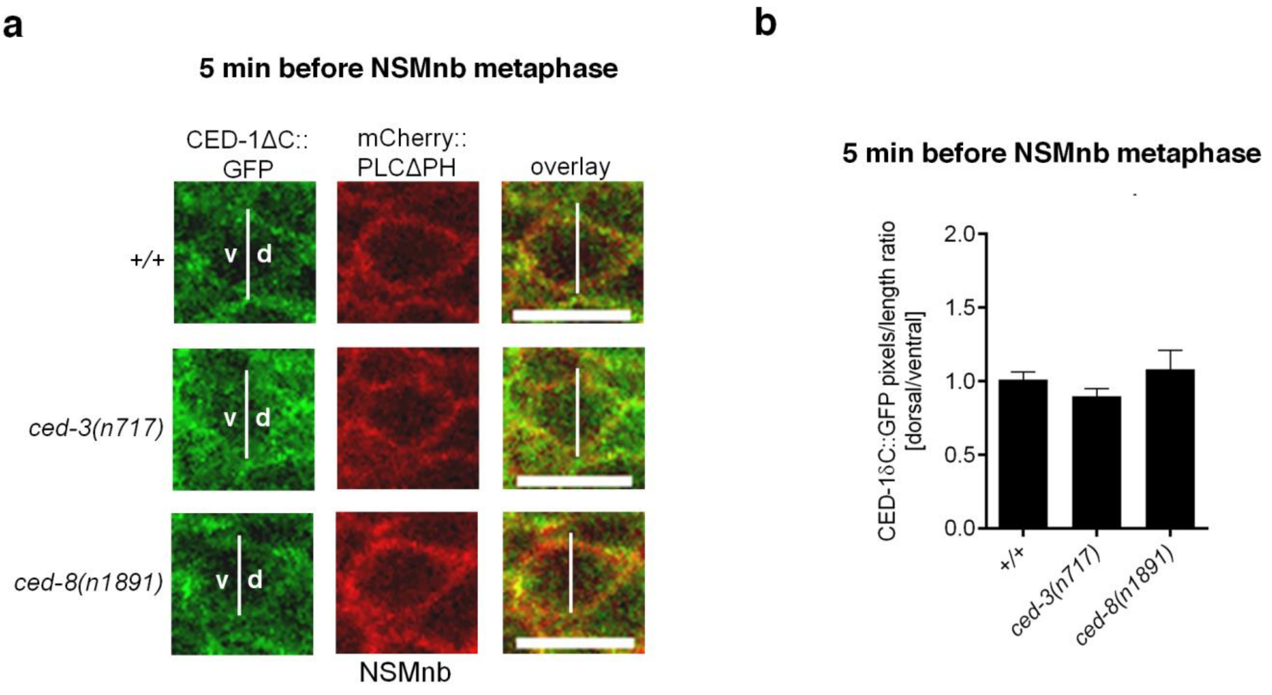

Supplementary Figure 5. Five min before metaphase, CED-1 is not enriched on neighboring cells apposing the dorsal side of the NSMnb.

a, Single channel and overlay projections of single plane confocal images of the NSMnb 5 min before metaphase in wild-type (+/+), *ced-3(n717)* and *ced-8(n1891)* embryos expressing $P_{ced\text{-}1}$*ced-1δC::gfp* (*enIs1*) and $P_{pie\text{-}1}$*mCherry::plcδph* (*ltIs44*). White vertical lines indicate the border between the dorsal 'd' and ventral 'v' side of the NSMnb (scale bar = 5 µm). **b,** Ratios of CED-1ΔC::GFP pixels/length apposing the dorsal and the ventral side of the NSMnb 5 min before metaphase in various genetic backgrounds (n=8-9). Statistics were performed using Student's t-test by comparing the respective means to wild-type (*p≤0.05, **p≤0.01 and ***p≤0.001). Error bars denote the standard error of the mean.

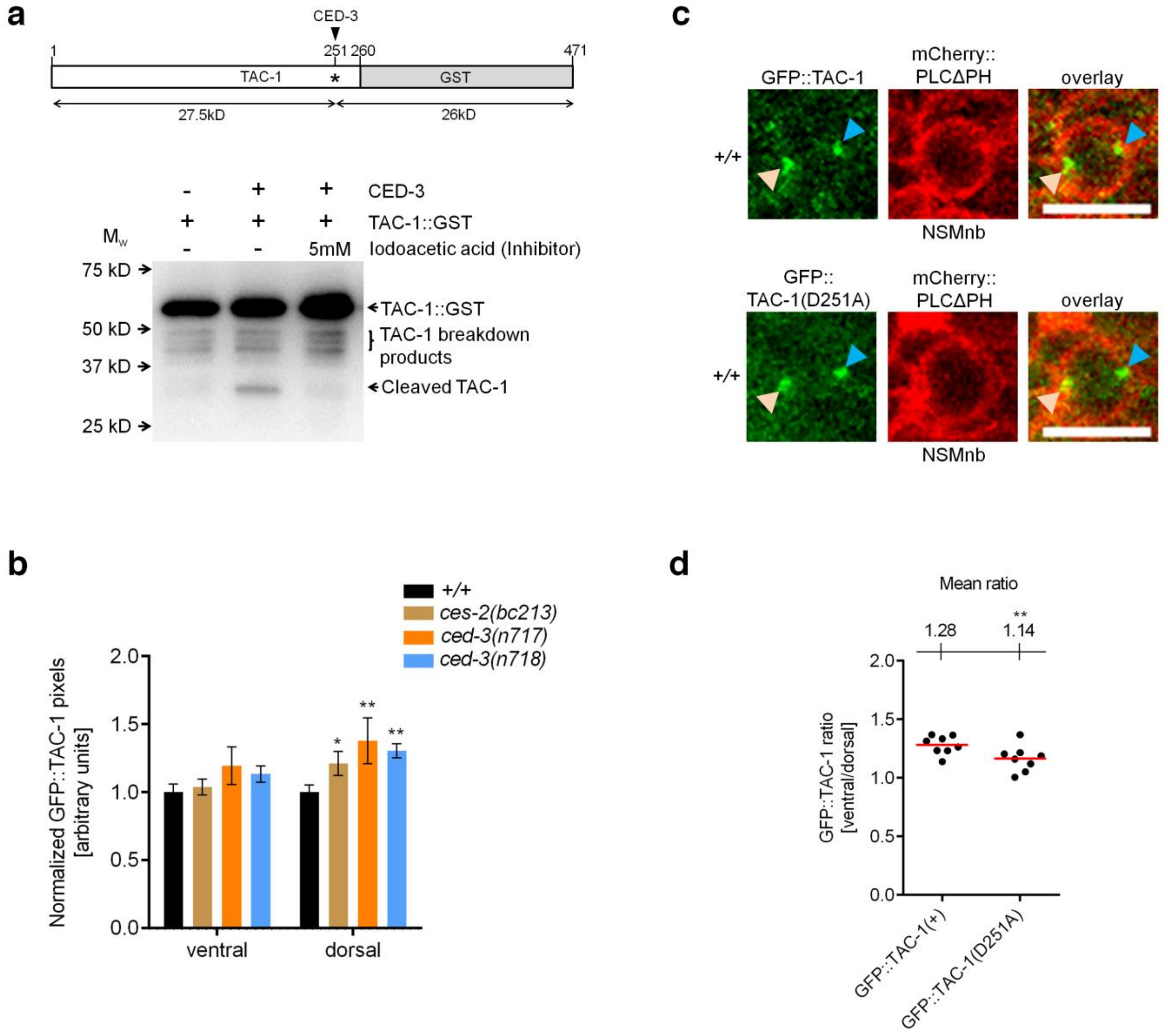

Supplementary Figure 6. TAC-1 is a substrate of CED-3.

a, Top. Schematic of TAC-1::GST fusion protein, which was used for CED-3 cleavage reactions *in vitro*. The CED-3 cleavage site in TAC-1 (*) as well as the expected cleavage products are indicated. Numbers above bar represent amino acid positions. Bottom: Western blot of different *in vitro* cleavage reactions. TAC-1::GST and TAC-1 were visualized using an antibody specific to *C. elegans* TAC-1. **b,** Normalized GFP::TAC-1 pixels [arbitrary units] associated with ventral or dorsal PCMs in the NSMnb at metaphase in wild-type (+/+), *ces-2(bc213), ced-3(n717)* and *ced-3(n718)* embryos expressing $P_{pie\text{-}1}$*gfp::tac-1* (*bcIs104*) and

$P_{pie\text{-}1}$*mCherry::plcδph* (*ltIs44*) (n=5-19). The mean pixel intensities of GFP::TAC-1 associated with the ventral or dorsal PCMs in wild-type (+/+) were set to 1 and the pixel intensities in *ces-2(bc213)*, *ced-3(n717)* and *ced-3(n718)* embryos were normalized against this wild-type value of 1. **c,** Maximum intensity single channel and overlay projections of confocal images of representative NSMnb at metaphase expressing $P_{pie\text{-}1}$*mCherry::plcδph* (*ltIs44*) and either $P_{pie\text{-}1}$*gfp::tac-1*(+) (*bcSi1*) (left) or $P_{pie\text{-}1}$*gfp::tac-1(D251A)* (*bcSi4*) (right) in wild-type (+/+) embryos (scale bar = 5 µm). Smaller insets represent enlarged projections of the two PCMs. Orange arrowheads indicate the PCM in the ventral part of the NSMnb and blue arrowheads indicate the PCM in the dorsal part of the NSMnb. **d,** Ratios of GFP::TAC-1 pixels associated with the ventral and dorsal PCM in individual NSMnb cells at metaphase in wild-type (+/+) embryos expressing $P_{pie\text{-}1}$*gfp::tac*-1(+) (*bcSi1*) (left) or $P_{pie\text{-}1}$*gfp::tac-1(D251A)* (*bcSi4*) (n=8). The mean ratio is given (top). Mean ratios were analyzed using the Student's t-test (*$p \leq 0.05$, **$p \leq 0.01$). Error bars denote the standard error of the mean.

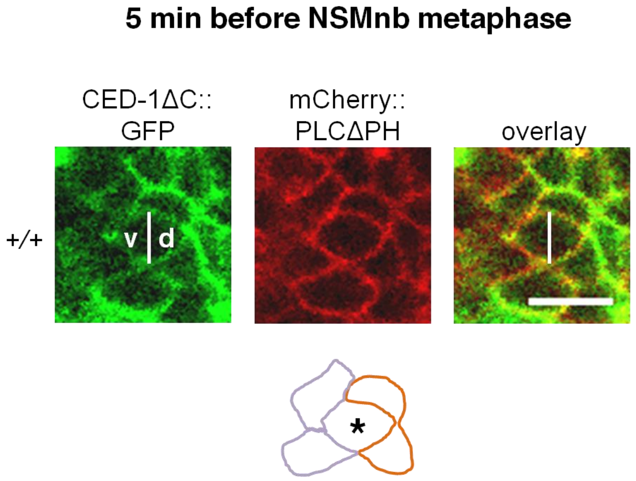

Supplementary Figure 7. CED-1 expression in dorsal and ventral neighbors of the NSMnb.

Single channel and overlay projections of a single plane confocal image of the NSMnb and its neighbors 5 min before metaphase in wild-type (+/+) embryos expressing P$_{ced\text{-}1}$*ced-1δC::gfp* (*enIs1*) and P$_{pie\text{-}1}$*mCherry::plcδph* (*ltIs44*). White vertical lines indicate the border between the dorsal 'd' and ventral 'v' side of the NSMnb (scale bar = 5 µm). Black asterisk in the schematic indicates the NSMnb. Orange and purple outlines indicate the cell boundaries of the dorsal and ventral NSMnb neighbors, respectively. mCherry::PLCΔPH (*ltIs44*) was used to visualize NSMnb cell boundary.

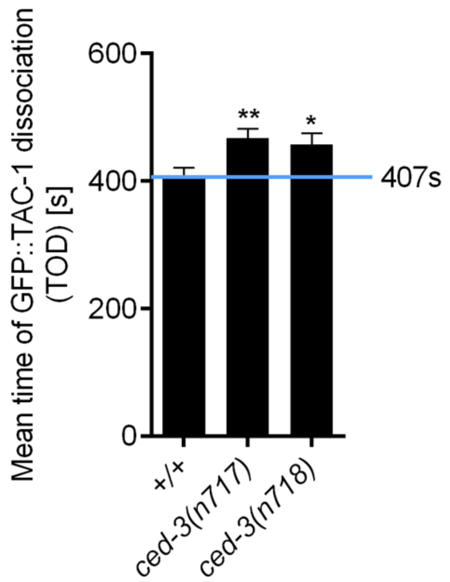

Supplementary Figure 8. CED-3 promotes the dissociation of GFP::TAC-1 from the PCM in the NSMsc.

Mean time of GFP::TAC-1 dissociation from the PCM (TOD) [s] segregated into the NSMsc in wild-type (+/+), *ced-3(n717)* and *ced-3(n718)*) embryos expressing $P_{pie\text{-}1}$*gfp::tac-1* (*bcIs104*) and $P_{pie\text{-}1}$*mCherry::plcδph* (*ltIs44*) (n=8-13). For comparison, the TOD in wild-type (+/+) is indicated by the blue line. Error bars denote the standard error of the mean. The mean times of dissociation were analyzed using the Student's t-test (*$p \leq 0.05$ and **$p \leq 0.01$). The statistical analyses were done in comparisons to wild-type (+/+).

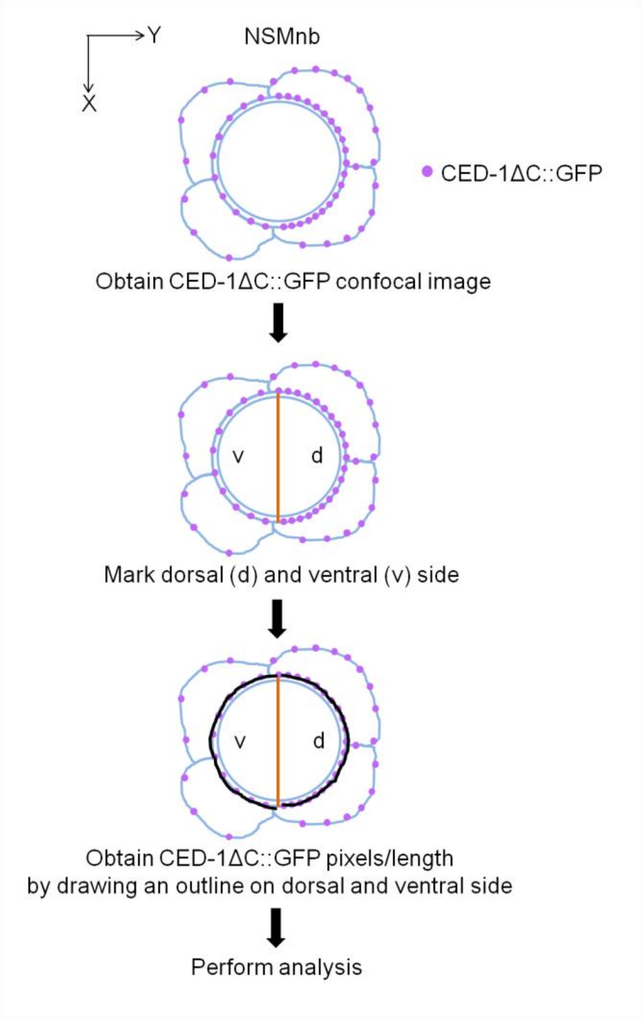

Supplementary Figure 9. Schematic description of CED-1ΔC::GFP analysis.

Confocal image of NSMnb, NSM and NSMsc were obtained as described (see Methods – Microscopy – Imaging of NSMnb). The dorsal (‘d’) and ventral (‘v’) sides were marked based on the division of the NSMnb. CED-1ΔC::GFP pixels/length were obtained by drawing on the dorsal and ventral sides using mCherry::PLCΔPH (*ltIs44*) as a reference and subsequent analysis was performed. For NSM and NSMsc, the same process was used but the complete cell boundary was taken into account (see Methods – Microscopy – CED-1ΔC::GFP analysis).

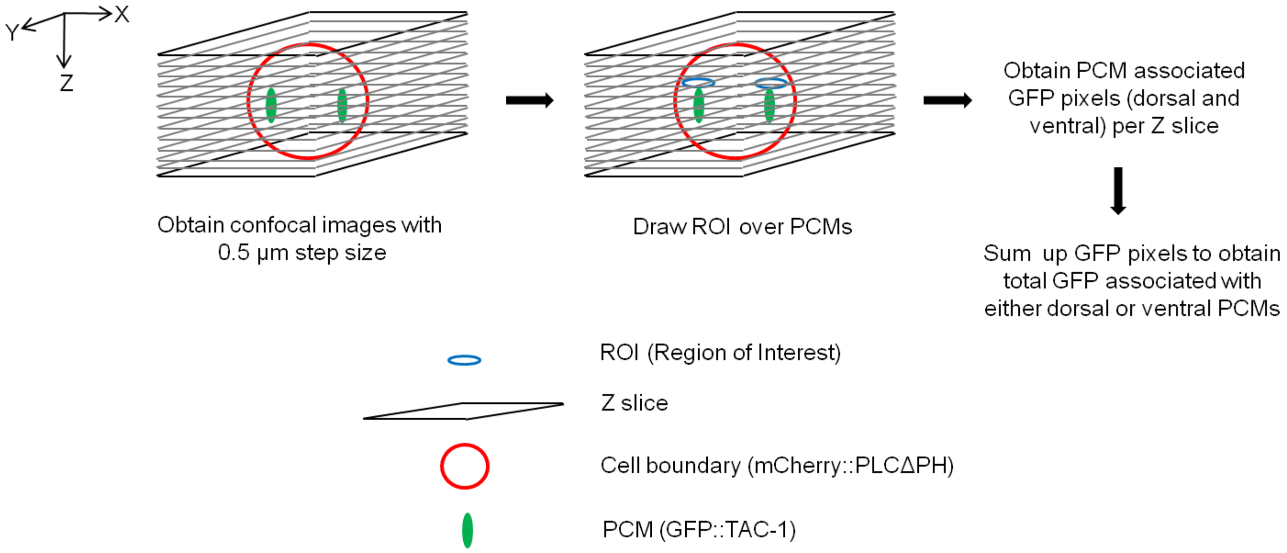

Supplementary Figure 10. Schematic description of the quantification of GFP::TAC-1 associated with dorsal and ventral PCM.

Confocal Z stack images as described in methods (see Methods – Microscopy – Imaging of NSMnb). A region of interest (ROI) was drawn around the dorsal and ventral PCM in the NSMnb [mCherry::PLCΔPH (*ltIs44*) was used to visualize NSMnb cell boundary], and the total GFP::TAC-1 pixels per PCM was obtained by adding up the numbers of GFP::TAC-1 pixels obtained for the individual Z slices as shown (see Methods – Microscopy – GFP::TAC-1 analysis).
